# Self-Regulation in the Time of Lockdown

**DOI:** 10.3389/fninf.2021.567920

**Published:** 2021-03-08

**Authors:** Anita Pacholik-Żuromska

**Affiliations:** Department of Cognitive Science, Institute of Information and Communication Research, Nicolaus Copernicus University in Toruń, Toruń, Poland

**Keywords:** outbreak, self-regulation, self, embodiment, enactivism, digital training tools

## Introduction

Since the beginning of the coronavirus disease 2019 pandemic, many studies have reported the psychological impact of the lockdown (mass quarantine). According to these studies, the societies affected are exposed to increased stress, mental tension, anxiety, and depression (Lee, 2020; Ozamiz-Etxebarria et al., 2020; Pandey et al., 2020; Sinnghal and Vijayaraghavan, 2020; Sugaya et al., 2020; Yuan et al., 2020). This paper aims to consider the possibility of minimizing the psychological effects resulting from the extraordinary situation caused by the outbreaks. I will propose an answer to the question of what methods will be effective in dealing with the negative psychological effects of lockdown and whether technological progress can benefit us in any way. In my opinion, effective results can be brought about by self-regulation methods based on biofeedback because they make it possible to develop the awareness of one's own body, reduce the feeling of detachment, and thus regain self-control (cf. Goessl et al., 2017). These methods are a good example of how the body affects the mind. The digital tools providing biofeedback are easy to use, so even people who are distrustful of digitalization can be convinced of their usefulness.

## Loss of Control

The emergence of abrupt emotional reactions during the outbreak of a pandemic is a natural phenomenon. It is an outcome of evolutionary bio-behavioral development (Tops et al., 2014). To survive in a given environment, an organism must adapt by minimizing the error in the prediction of the possible state of the world (Friston, 2009, 2012). The adaptation then would be seen as a disposition toward the avoidance of an informational surprise emerging from the environment (Friston, 2009, 2012). The lower the entropy, the higher the predictability of the environment. Although the prediction error is a necessary element of learning (Joiner et al., 2017), if it occurs too often, a low, predictable environment is created, which causes reactive behavior involving strong emotions as an adaptive response (Tops et al., 2014): acting according to the direct stimuli, the involvement of exogenous attention, associative learning (Tops et al., 2014), and minimal rationality, indicating an action that is of maximal use for an agent, regardless of the consequences for others (Cherniak, 1981). On the other hand, a highly predictable environment causes proactive behavior, which is less affective and allows rational coping and self-reflection (Tops et al., 2014).

In my opinion, outbreaks and their social consequences create an abnormal situation, indicating a low, predictable environment in which, for a long time, individuals cannot function properly. Everyday reference points, such as actions and undertakings, owing to which we maintain balance and which define us and keep us in line, are fuzzy and distorted. What constitutes the ground for such negative psychological reactions is the disintegration of the self, which occurs due to the loss of the locus of control.

The evidence of this claim can be found, for example, in studies on disorders of the self, such as autism spectrum disorder and schizophrenia. The locus of control, which means the self, is here disturbed by either a too weak or too strong “bodily boundary between self and other” (Noel et al., 2017, p. 1). In these cases, the therapies changing the self-representation by altering the experience of self-location by using “synchronous administration of spatially fixed exteroceptive (i.e., visual and/or auditory) sensory signals with tactile information” were proposed (Noel et al., 2017, p. 8). Such therapies, in my opinion, fall under the category of therapies with biofeedback, which is what I mean here. Thus, not only in the time of lockdown but also in other situations, there is a need for a professional tool to help restore a sense of control and bring it back to the self through the body.

## Self-Regulation and Biofeedback

One of the ideas of how to deal with a low, predictable environment is to build resilience while replacing reactive behavior with proactive behavior (Dehnad, 2017) to respond less emotionally and more confrontationally to an abnormal situation. I see such potential in a digital training of self-regulation with biofeedback.

Self-regulation has many dimensions. It can be understood in a physiological sense as the maintenance of homeostasis and “compensatory responses to the discrepancy between a system's reference state and its input stage” (Jeannerod, 1993, p. 83, see also Jeannerod, 2006). In a psychological sense, self-regulation refers to emotions and suggests “initiation or alteration of ongoing emotional responses through cognitive processes” (Heatherton, 2011, p. 375). Additionally, society fulfills a regulative role when a subject compares his or her perspective with the perspective of others and modifies his or her attitudes to conform to the norms (Tomasello, 1993, 2019). In terms of biofeedback, self-regulation is understood as a result of combining the biological and psychological dimensions. In such an account, self-regulation increases the sense of control over one's own body by engaging in proprioceptive and attentional training (Cf. Blanke and Metzinger, 2009). In general, biofeedback indicates information about the state and condition of one's own body provided by various measurement tools (Schwartz and Andrasik, 2017). The purpose of biofeedback is to enhance bodily awareness to consciously and intentionally regulate physical states such as muscle tension or heart rate. To achieve this ability, the psychophysical (internal–external) coupling in self-representation should be strengthened.

In the understanding of self-regulation proposed here, it is clear that enhancing the connection with one's own body will lead to the restoration of self-control. Therefore, there is a need for innovative digital tools offering modern psychological healthcare to regulate the embodied and enactive self and thereby increase psychological resilience and sustain mind–body balance.

## Digitalization of Self-Regulation

There are already many studies on using digital technologies to train various social competencies (Gaggioli et al., 2019). Mobile applications dedicated to self-regulation often refer to the elements of mindfulness, such as focused attention and hand–eye coordination (Tang et al., 2007). These tools are quite interesting because they provide higher motivation for training owing to their gamification. The engagement of attention and the senses of vision and touch regulate proprioception (Gibson, 2002), although the influence on proprioception by mobile applications is far smaller than by virtual reality (VR) (Lenggenhager et al., 2007; Blanke et al., 2015). The mobile apps have other disadvantages because they do not give biofeedback and strain visual attention owing to the need to focus on the display. They are also sometimes immensely complicated for people less familiar with new technologies. More importantly, in a demanding situation, such as a pandemic, such people will not use tools that additionally stress them. Therefore, there is a need to develop devices that help in self-regulation in a low, predictable environment and offer training support to cope with stress in situations that go beyond ordinary everyday life.

The idea is then to adapt digital tools to the specific conditions governing the limitations of daily activities. Such digital devices for self-regulation training should involve movement and give biofeedback. These applications do not seem possible on one-piece hardware such as a mobile phone. Rather, there is a need for separate sensors and a device for collecting data and giving feedback on this basis. These sensors could also be the elements of training. For example, they could be interactive marbles to hold on open hands.^1^ One of the training tasks would be to perform body movements to keep balance and not drop the marbles. They could also be interactive rings on fingers. There are plenty of possibilities. The point is that the sensor placed on body parts, such as arms, fingers, and legs, would collect the data from, for example, electrodermal activity, muscle tension, and pulse and send them to the control unit in a separate device. This device could be similar to the usual fitness watch, although with a voice guide (a kind of assistant) giving instructions according to the data to adjust the user movement. Important here is the calibration and personalization of the device. The advantage of such a tool would be that it requires movement and, at the same time, gives feedback without having to look at the display, and thereby it does not strain the sense of vision.

The constant need to create and develop such digital tools was recognized by Colombo et al. (2019), especially with reference to emotion regulation. The authors extensively reviewed the technologies from the internet-based interventions *via* mobile health to virtual reality, which they perceived as good support for self-regulation. Among them, they also placed biofeedback. They then diversified the biofeedback from other forms and also proposed mixed forms, for example, VR with biofeedback (Colombo et al., 2019). I can only support this idea; although I see the limits of its use by older generations, first because of their possible aversion to new technologies and second because of an increased probability of motion sickness (Lee et al., 2017). At this juncture, VR also faces some substantial limits in user experience, such as a heavy head-mounted display or wiring. Nevertheless, VR offers a variety of environments where, owing to strong immersion, a subject feels like he/she is in a real situation, giving many possibilities of training by action. It is also worth mentioning another reason for the combination of gamification and biofeedback—the necessity of self-regulation during or even before playing (Seay and Kraut, 2007). My argument for this need is that sometimes subjects are not able to recognize that they need self-regulation because bodily alarming signals do not reach the field of consciousness or subjects cannot identify emotions, which are too fuzzy (Schooler and Schreiber, 2004). This happens, in my opinion, in those situations that cause reactive behavior, lacking self-reflection, and, hence, self-regulation. Under such circumstances, the use of gamified tools in the hope of reacting to stress can have even negative effects such as addiction (Seay and Kraut, 2007). In such deficits, the biofeedback delivers information about the physical state of the body—which correlates with the psychological state—before the subject realizes (if at all) that their current emotions are overwhelming them.

## Conclusion

It can be said that the coronavirus disease 2019 pandemic situation has forced us to speed up the progress in digitalization. There is a sudden need to create tools that help in self-regulation and thus support coping with anxiety caused by the unusual stressor—the outbreak. At the same time, and unlike other digital tools in healthcare, it is difficult to define to whom such devices would be dedicated because the target is highly differentiated according to factors, including age, sex, and character—that is why such tools need to be easily calibrated and personalized. Furthermore, as has been emphasized, digital training in self-regulation cannot be an additional psychological burden, but on the contrary, it must release positive emotions; otherwise, no one will use it more than once. Finally, from a philosophical viewpoint, such tools are an example of “the extended mind” (Clark and Chalmers, 1998) as they improve self-reflection and help in self-cognition when it starts to fail in a stressful situation and when reactive behavior is triggered. Thus, they allow one to regain self-control and rebuild the connection to oneself.

## Author's Note

AP-Z refers in the manuscript to the mobile application Stabilo, created by Neurodio LLC and Kogni_LAB. The Lab is a unit of the Department of Cognitive Science chaired by AP-Z. AP-Z was also an initiator of the project Stabilo.

## Author Contributions

AP-Z confirms sole responsibility for the conception, study, and manuscript preparation.

## Conflict of Interest

The author declares that the research was conducted in the absence of any commercial or financial relationships that could be construed as a potential conflict of interest.

## Footnotes

^1^In the laboratory belonging to the Department of Cognitive Science, a team of scientists, together with Neurodio LLC, has created a relaxing application *Stabilo*, in which the idea of a balancing marble was also used. https://play.google.com/store/apps/details?id=com.Neurodio.Stabilo. The aim of the application is to train mindfulness as a method of coping with stress. From my perspective, what we learn from *Stabilo* first is patience because, at the beginning, it is extremely hard to balance with the virtual marble, but once we manage it, we can easily deal with any other kinds of stress.
